# High-Resolution OFDR with All Grating Fiber Combining Phase Demodulation and Cross-Correlation Methods

**DOI:** 10.3390/s26031004

**Published:** 2026-02-03

**Authors:** Yanlin Liu, Yang Luo, Xiangpeng Xiao, Zhijun Yan, Yu Qin, Yichun Shen, Feng Wang

**Affiliations:** 1 Key Laboratory of Intelligent Optical Sensing and Manipulation (MoE), Nanjing University, Nanjing 210023, China; 502024340009@smail.nju.edu.cn (Y.L.); 522025340020@smail.nju.edu.cn (Y.L.); 2 Nanjing University (Suzhou) High-Tech Institute, Suzhou 215000, China; 3 School of Optical and Electronic Information, Huazhong University of Science Technology, Wuhan 430074, China; xpxiao@hust.edu.cn (X.X.); yanzhijun@hust.edu.cn (Z.Y.); 4 Advanced Fiber Devices and Systems Group, Key Laboratory of Micro and Nano Photonic Structures (MoE), College of Future Information Technology, Fudan University, Shanghai 200433, China; 22110720122@m.fudan.edu.cn (Y.Q.); shenyc@ztt.cn (Y.S.); 5 Zhongtian Technology Advanced Materials Co., Ltd., Nantong 226009, China

**Keywords:** OFDR, phase demodulation, cross-correlation, strain sensing

## Abstract

Spatial resolution is a critical parameter for optical frequency domain reflectometry (OFDR). Phase-sensitive OFDR (Φ-OFDR) measures strain by detecting phase variations between adjacent sampling points, having the potential to achieve the theoretical limitation of spatial resolution. However, the results of Φ-OFDR suffer from large fluctuations due to multiple types of noise, including coherent fading and system noise. This work presents an OFDR-based strain sensing method that combines phase demodulation with cross-correlation analysis to achieve high spatial resolution. In the phase demodulation, the frequency-shift averaging (FSAV) and rotating vector summation (RVS) algorithms are first employed to suppress coherent fading noise and achieve accurate strain localization. Then the cross-correlation approach with an adaptive window is proposed. Guided by the accurate strain boundary obtained from phase demodulation, the length and position of the cross-correlation window are automatically adjusted to fit for continuous and uniform strain regions. As a result, an accurate and complete strain distribution along the entire fiber is finally obtained. The experimental results show that, within a strain range of 100–700 με, the method achieves a spatial resolution of 0.27 mm for the strain boundary, with a root-mean-square error approaching 0.94%. The processing time reaches approximately 0.035 s, with a demodulation length of 1.6 m. The proposed approach offers precise spatial localization of the strain boundary and stable strain measurement, demonstrating its potential for high-resolution OFDR-based sensing applications.

## 1. Introduction

OFDR has been widely used due to its high spatial resolution [1]. This technique has been extensively employed in pipeline monitoring [2,3], structural health monitoring [4,5], shape sensing [6,7,8] and biomedical applications [9,10,11]. Meanwhile, applications in microstructure inspection and precision measurement impose more stringent requirements on the spatial resolution of OFDR. The OFDR technique can be categorized into phase demodulation and cross-correlation demodulation methods. Among them, Ф-OFDR achieves higher spatial resolution compared with the amplitude cross-correlation algorithms [12].

However, in Φ-OFDR systems, the phase demodulation results usually exhibit pronounced fluctuations [13,14]. This is mainly attributed to noise, which becomes evident during the phase demodulation process. Such noise is the combined effect of multiple noise sources, including coherent fading induced by Rayleigh scattering as well as other system noise [15]. These noise sources are superimposed during phase extraction, which eventually causes the demodulated phase of Φ-OFDR to exhibit large fluctuations.

To address the problems caused by noise, several approaches have been proposed [16,17,18]. Widely used methods include filtering-based approaches and phase correction techniques.

In filtering-based approaches, various signal processing strategies, such as the median filter [19] and the Savitzky–Golay digital differentiator [14], are employed to filter noise, thereby improving the stability of phase demodulation [20]. However, these methods often lead to a significant degradation in spatial resolution, in contradiction with the high-spatial-resolution demands of using Φ-OFDR.

In terms of phase correction methods, Zhao et al. proposed a phase retrieval method based on density distribution with segment-wise phase correction [21]. Specifically, phase correction is achieved through linear phase compensation applied to each segment. Nevertheless, the demodulated phase still exhibits fluctuations with an amplitude of approximately 1 rad, leading to a strain resolution in the order of several hundred με at a spatial resolution of 1 mm. This indicates an inherent trade-off between spatial resolution and strain resolution. Building upon this framework, Lv et al. proposed a Φ-OFDR method based on the position mismatch compensation–outlier phase correction scheme [22]. In this method, phase correction is applied to the phase information of the reference and measurement signals to restore phase coherence and improve the reliability of phase demodulation. This approach achieved a strain resolution of 4 με, while the spatial resolution was limited to 23.2 mm. Therefore, although the noise is effectively suppressed, the trade-off is not fundamentally eliminated, but rather, shifted. In addition, this algorithm is relatively complex and typically involves multiple threshold parameters for decision making, which need to be retuned under different experimental conditions. This increases the difficulty of practical implementation in real engineering applications.

In contrast to phase demodulation methods, cross-correlation methods represent another important demodulation route in OFDR. These methods exhibit high strain stability and strong noise immunity. However, their spatial resolution is inherently limited by the correlation window length [23].

To overcome the above limitations, this paper proposes a strain demodulation method that combines cross-correlation with phase demodulation methods for distributed strain measurements along a 1.6 m long all grating fiber (AGF, Huazhong University of Science and Technology, Wuhan, China). The method exploits the point-by-point calculation capability of phase demodulation to achieve high spatial resolution. The FSAV and RVS methods are employed to mitigate coherent fading noise. Therefore, the strain boundaries are accurately identified. Based on this, an adaptive cross-correlation window is introduced. The window is dynamically guided by the phase-derived strain boundaries and is confined within a single strain level. In this way, a phase-guided adaptive correlation framework is formed. The entire fiber is first divided into multiple continuous strain segments according to the detected strain boundaries. The start and end positions of the sliding window are adjusted according to the identified strain boundaries, ensuring that each window covers only a single continuous strain region. As a result, distortion and multi-peak structures of the main correlation peak caused by mixing signals from different strain levels within the same window are effectively avoided.

By integrating the strain information derived from both phase demodulation and cross-correlation, the proposed method achieves precise spatial localization and quantitative evaluation of strain. The experimental results demonstrate that the proposed scheme exhibits significant advantages in both measurement accuracy and noise suppression.

## 2. Measurement Principle and Experimental Setup

### 2.1. Measurement Principle

**Figure 1:**
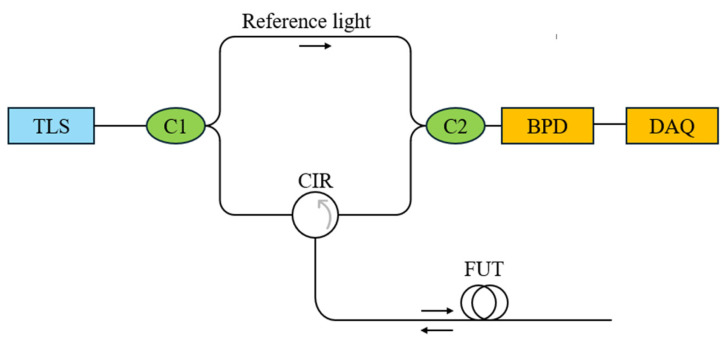
OFDR sensing principle. TLS: tunable laser source; C1, C2, optical couplers; CIR, optical circulator; FUT: fiber under test; BPD: balanced photodetector; DAQ: data acquisition.

Figure 1 illustrates the sensing principle of the OFDR system. A tunable laser source generates a linear frequency sweep, and coupler C1 splits this light into reference and measurement arms. The light in the measurement arm is launched into the sensing fiber, and the returning Rayleigh backscattering signal (RBS) or reflected light interferes with the reference light at coupler C2, generating a beat signal. This beat signal, corresponding to a location *z* in the fiber, is given by [24,25]:

(1) $$I(t)=2E_{0}^{2}\sqrt{R(\tau )}\cos[2\pi (\gamma \tau t+v_{0}\tau -\frac{1}{2}\gamma \tau^{2})]$$

where I(t) is the beat signal, $E_{0}$ is the signal amplitude, R(τ) is the reflection coefficient, τ=2nz/c is the delay time at position *z*, $v_{0}$ is the initial optical frequency, and γ is the sweep rate of the light source. Performing a fast Fourier transform (FFT) on the above equation, the extracted phase term can be expressed as [20]:

(2) $$\phi =2\pi (v_{0}\tau -\frac{1}{2}\gamma \tau^{2})$$

Since $v_{0}\tau \gg \frac{1}{2}\gamma \tau^{2}$, the equation can be rewritten as:

(3) $$\phi (z)=\frac{4\pi nv_{0}z}{c}$$

The measurement of strain for OFDR requires the acquisition of two signals before and after the strain, which are referred as the reference signal and measurement signal respectively. These signals are generated when the laser wavelength is tuning, so we refer them as spectral-domain signals. After applying FFT to each signal, distance-domain signals are obtained. From these signals, the corresponding phase spectra along the fiber can be further extracted.

Based on the phase spectra, strain demodulation can be performed via phase demodulation. The phase difference induced by the strain can be expressed as [26]:

(4) $$\Delta \phi (z)=\frac{4\pi }{\lambda_{0}}[n_{eff}\Delta L(z)+\Delta nL(z)]$$

where $\lambda_{0}$ is the initial wavelength, $n_{eff}$ is the effective refractive index, L(z) is the length of the strained region, Δn is the change in refractive index, and ΔL is the change in fiber length. Considering the refractive index variation originating from the elasto-optical effect [27], we can obtain:

(5) $$\frac{\Delta n}{n_{eff}}=-P_{e}\varepsilon$$

Substituting (5) into (4) and differentiating with respect to *z*, we can obtain:

(6) $$\frac{diff[\Delta \phi (z)]}{\Delta z}=\frac{4\pi n_{eff}(1-P_{e})}{\lambda_{0}}\frac{diff[\Delta L(z)]}{\Delta z}$$

where diff refers to the numerical difference between adjacent data points. Therefore, the strain distribution along the optical fiber can be expressed as:

(7) $$\varepsilon =diff[\Delta \phi (z)]\frac{1}{\Delta z}\frac{\lambda_{0}}{4\pi n_{eff}(1-P_{e})}$$

According to Equation (7), the strain is linearly proportional to the differential phase difference along the sensing fiber. Therefore, the localization of the strain boundary can be determined by analyzing variations in the differential phase difference. The spatial resolution Δz=c/(2nΔF) is determined by the laser frequency sweep range ΔF. However, phase demodulation is highly sensitive to noise, resulting in large fluctuations and reduced strain measurement accuracy.

In contrast, the cross-correlation method is generally robust to noise, providing more stable strain measurements. The local reference and measurement segments consisting of N points are chosen from the distance-domain signals. Subsequently, an inverse FFT (IFFT) is applied to the local reference and measurement segments to obtain the local corresponding spectral-domain signals. The key to strain calculation using the cross-correlation method lies in extracting the spectral shift between local spectral-domain signals. The relationship between the spectral shift m and the strain ε can be expressed as [23]:

(8) $$\varepsilon =\frac{m\Delta F}{NS}$$

where N represents the number of sampling points contained in the correlation window, and S is the strain sensitivity coefficient. The spectral shift m manifests as a displacement of the main peak in the cross-correlation spectrum. Therefore, the peak shift can be obtained by performing cross-correlation between the two spectra, which enables strain calculation along the fiber. The cross-correlation method offers high strain stability and strong noise immunity. However, its spatial resolution is limited by the window width.

**Figure 2:**
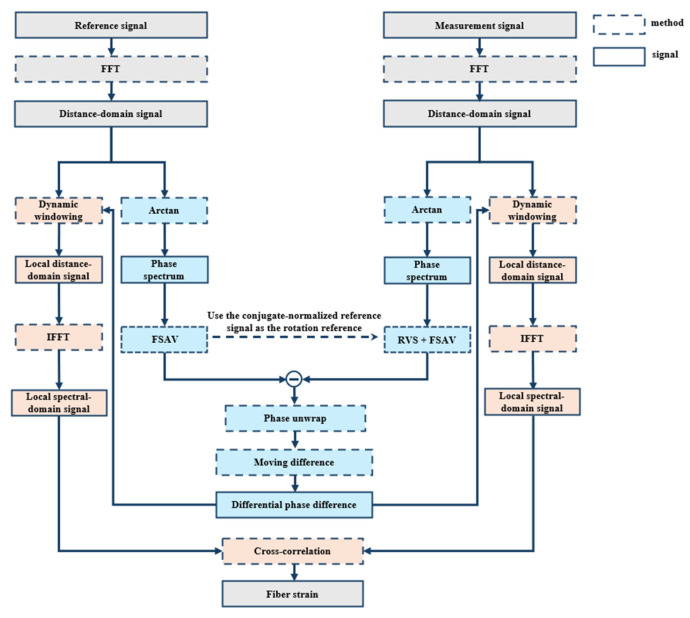
Flowchart of fiber strain calculation via combined OFDR phase demodulation and cross-correlation.

To address the inherent trade-off between spatial resolution and strain resolution, phase demodulation and cross-correlation analysis are jointly employed in this work. The measurement procedure of the proposed method is illustrated in Figure 2, followed by a detailed description of each processing step.

First, the OFDR sensing system acquires a set of spectral-domain signals as the reference signals before strain is applied on the FUT, and then acquires another set of spectral-domain signals as the measurement signals after the strain. The phase spectra are obtained from the distance-domain signals after their FFT.

Subsequently, the extracted phase spectra are processed using a combined FSAV and RVS algorithm to suppress coherent fading noise [28]. First, the RVS algorithm is incorporated to rotate the distance-domain measurement signal prior to summation. Specifically, a baseline vector $\vec{r_{b}}=\frac{\vec{r_{r}}}{r_{r}}$ is constructed by conjugate normalization of the reference signal, and the measurement signal is then rotated point by point toward the direction of the baseline vector. Through this rotation operation, random phase fluctuations induced by coherent fading noise are effectively suppressed, while the strain-induced phase components are largely preserved. After completing the rotation, the rotated results obtained via FSAV are summed and averaged. As a result, the signal amplitude is not reduced during averaging, and coherent fading noise is effectively mitigated.

To obtain the distributed strain, a sliding window is applied along the distance-domain signals to extract local segments from the reference and measurement signals. Each segment is transformed back into the spectral domain via IFFT, and cross-correlation between the two corresponding spectral-domain segments is computed to determine the peak shift, from which the local strain is derived. By continuously sliding the window along the entire distance-domain signal, the full distributed strain profile is obtained.

However, when a correlation window crosses a strain boundary, signals from different strain areas are mixed within the same window, leading to distortion and multiple peaks, deteriorating the measurement results. To address this issue, an adaptive cross-correlation window strategy is introduced. Guided by the accurately localized strain boundaries obtained from phase demodulation, the length of the correlation window is dynamically adjusted to keep entirely within the same strain region. Through this approach, accurate and complete distributed strain measurement along the sensing fiber is achieved.

Phase demodulation and cross-correlation methods exhibit complementary characteristics. Phase demodulation provides high spatial resolution for accurately locating the strain boundary. This alleviates the limitation of spatial resolution imposed by the window width in the cross-correlation method. Meanwhile, cross-correlation is more robust and can mitigate the sensitivity of phase demodulation to noise.

Noise in the phase signal causes large fluctuations. This makes it difficult to identify abrupt strain boundaries from the phase gradient. The proposed adaptive correlation window uses the boundary information. When the correlation window crosses a strain boundary, the main correlation peak is distorted, leading to demodulation errors. Based on these features, the proposed method forms a feedback mechanism. First, the high-resolution phase information is used to locate the strain boundary. Then, the cross-correlation window is adaptively confined within a single strain level. This avoids the distortion of the main correlation peak caused by windows crossing the boundary and leads to stable and reliable correlation results.

### 2.2. Experimental Setup

**Figure 3:**
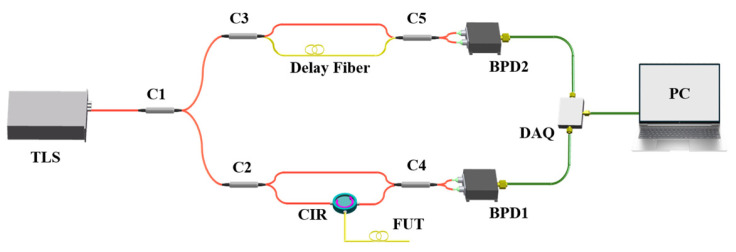
Schematic of the experimental setup. PC: computer.

Figure 3 illustrates the experimental setup of a phase-sensitive OFDR-based strain sensing system. The light source is a tunable laser (Phoenix 1400, Luna Innovations Inc., Roanoke, VA, USA) with a wavelength sweep range of 1535–1565 nm and an output power of 7 dBm.

The swept light is divided by coupler C1 (splitting ratio 90:10) into two paths: 10% is routed to the auxiliary interferometer, and the remaining 90% is introduced into the main interferometer. Both interferometers are constructed in a Mach–Zehnder configuration. The coupler C2 (90:10) splits the light entering the main interferometer into the measurement light (90%) and the reference light (10%). The measurement light is directed through CIR into the FUT, while the reference light is combined with the returning measurement light in coupler C4 (50:50). This combined signal is converted by BPD1 (DC-80 MHz bandwidth, Aoshow Information Technology Co. Ltd., Shanghai, China) and digitized by the DAQ (FC9825, Fcctec Technology Co. Ltd., Beijing, China). Ten percent of the swept light is divided by coupler C3 (50:50) into reference and delay paths. These two light beams then interfere at coupler C5 (50:50), generating a beat signal with a frequency of 23.55 MHz. This signal is converted into an electrical signal by BPD2 (DC-300 MHz bandwidth, Aoshow Information Technology Co. Ltd., Shanghai, China) and serves as the external clock for the DAQ. Furthermore, AGF with a total length of 1.6 m is employed as the FUT to enhance the signal-to-noise ratio (SNR).

## 3. Results and Discussion

### 3.1. Amplitude Profile of the Distance-Domain Signal

**Figure 4:**
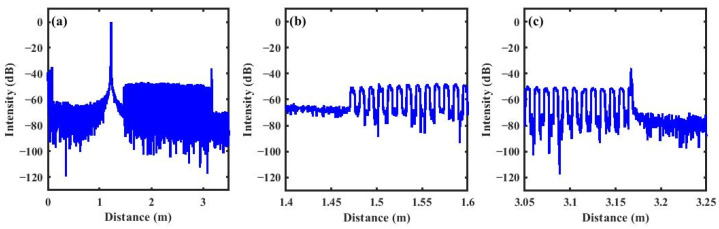
(**a**) Distance-domain amplitude profile of the entire FUT; (**b**) distance-domain amplitude profile at the start of the FUT; (**c**) distance-domain amplitude profile at the end of the FUT.

After performing a FFT on the measurement signal, the distance-domain amplitude signal along the sensing fiber is obtained by calculating the magnitude of the Fourier-transformed signal, as shown in Figure 4. Periodic dips with a 10 mm period are observed, which corresponds to the alternating structure of the FUT with a grating length of 5 mm and a grating spacing of 5 mm. The reflective signal in the grating sections exhibits a signal intensity approximately 20 dB higher than the RBS in the grating spacing sections. Thus, the signal from the grating spacing sections exhibits a relatively high amount of noise, which leads to abrupt phase jumps and causes errors in phase demodulation. The FSAV/RVS algorithm suppresses coherent fading noise over the entire signal. It improves the performance in the dip sections and enhances the overall phase stability. Therefore, the FSAV/RVS step is important.

### 3.2. Strain Measurements

**Figure 5:**
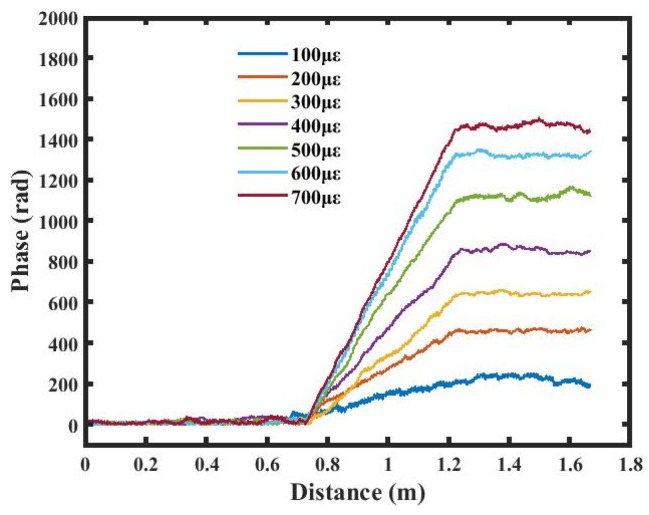
Unwrapped phase curves under different strains.

After performing an FFT on both the reference and measurement signals, the corresponding phase components are extracted and subtracted to obtain the phase difference, followed by phase unwrapping. As shown in Figure 5, the unwrapped phase curves of the FUT can be obtained under different strain conditions. In the strained regions, the fiber phase increases approximately linearly with the fiber length, and a larger strain corresponds to a steeper slope of the phase curve, indicating a proportional relationship between phase variation and local strain along the fiber.

In the strain-free regions, the phase generally remains stable; however, due to noise, abrupt jumps occur frequently in the phase curve, leading to fluctuations in the demodulated results.

**Figure 6:**
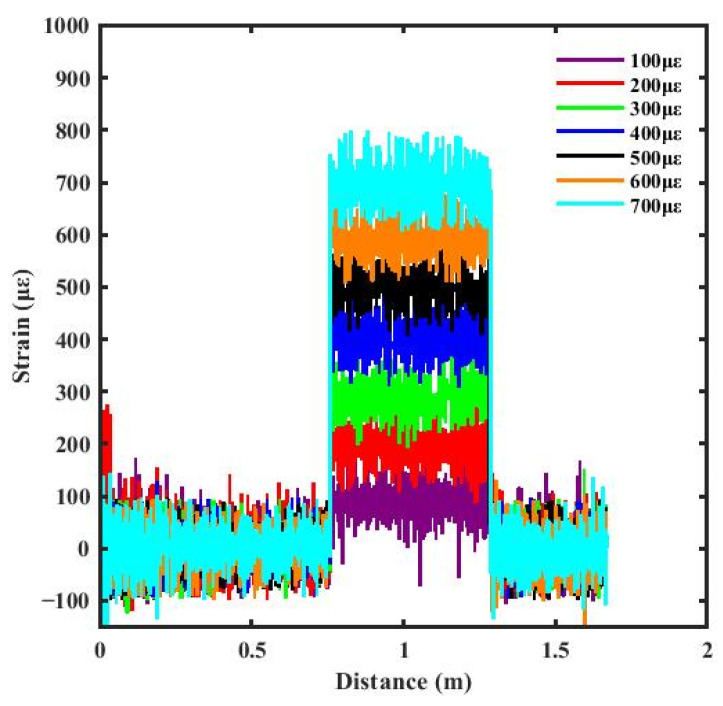
Strain results from phase demodulation only.

According to Equation (7), the strain distribution of the FUT can be obtained by numerically differentiating the unwrapped phase along the fiber length. As shown in Figure 6, the resulting strain curve exhibits significant random fluctuations. This result is attributed to the sensitivity of the phase demodulation method to various types of noise, which is further amplified by the numerical differentiation process.

**Figure 7:**
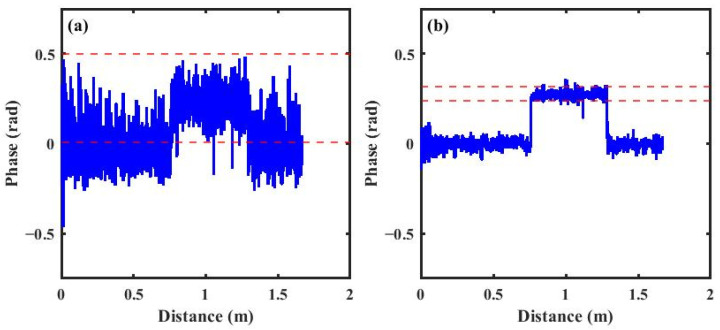
(**a**) Differential phase difference before RVS; (**b**) differential phase difference after RVS.

Therefore, denoising and filtering algorithms are commonly employed in practical applications to improve the accuracy and reliability of strain measurements. By combining FSAV and RVS algorithms, coherent fading noise can be effectively suppressed, thereby improving the accuracy of the phase demodulation method. Figure 7a shows the differential phase difference before applying the FSAV and RVS algorithms, reflecting the noise. In the RVS algorithm, a window overlap ratio of r = 0.5 and a window width of w = 60 were used, and both the reference and measurement signals were divided into 205 segments for analysis. For each reference signal segment, a normalized conjugate vector was constructed and multiplied with the corresponding measurement signal segment to obtain the rotated measurement signal segment. The rotated measurement signal segments were then summed and averaged, and the phase difference was obtained by subtracting the averaged reference signal segments, followed by phase unwrapping and differential calculation. The RVS algorithm effectively improves the stability and reliability of the differential phase difference.

Figure 7b presents the results after subsequently applying the FSAV and RVS algorithms. It can be clearly observed that the phase fluctuations in the strained regions are effectively suppressed, with the maximum amplitude reduced by approximately 0.4 rad. The number of abrupt jumps in the curve significantly decreased. This improvement not only enhances the smoothness of the differential phase difference but also improves the accuracy of strain boundary localization.

**Figure 8:**
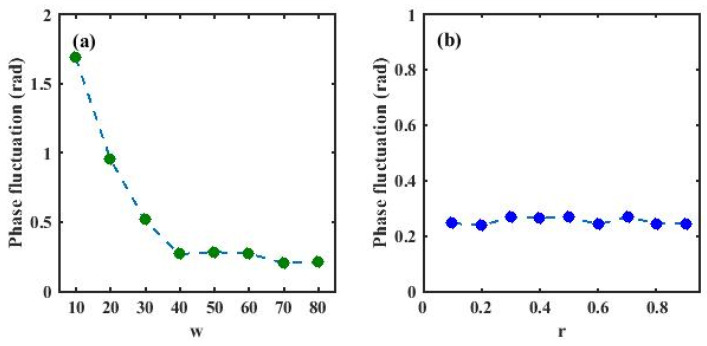
(**a**) Dependence of phase fluctuation on w with r fixed; (**b**) dependence of phase fluctuation on r with w fixed.

As shown in Figure 8, it can be observed that when r = 0.5, increasing w from 10 to 40 significantly reduces the phase fluctuation. For larger values of w, the value of phase fluctuation tends to level off. When w = 60, varying r from 0.1 to 0.9 nearly does not affect the phase fluctuation, indicating that the phase fluctuation is weakly sensitive to r.

**Figure 9:**
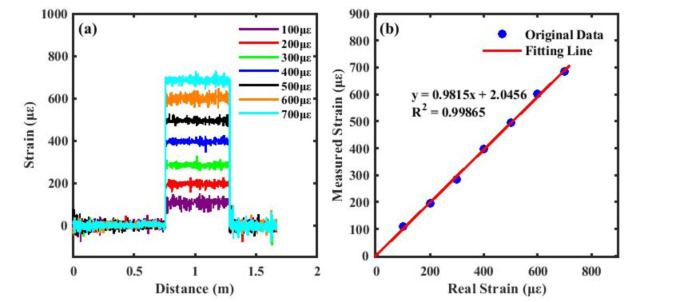
(**a**) Different strain measurements under phase demodulation; (**b**) linear fit between actual and measured strains.

To achieve higher phase measurement accuracy within the experimental strain range, the laser in this study was configured with an effective frequency sweep range of 0.37 THz and a sweep rate of 70 nm/s, corresponding to a theoretical spatial resolution of approximately 0.27 mm. Phase demodulation was performed within a strain range of 100–700 µε to ensure the acquisition of stable and precise strain distributions. As shown in Figure 9a, after applying the FSAV and RVS algorithms, individual strain values obtained through phase demodulation can be clearly distinguished, indicating that the adopted algorithms can effectively suppress noise. Figure 9b further illustrates a highly consistent linear relationship between the actual and measured strain values, with the entire measurement process exhibiting excellent reliability. The obtained correlation coefficient of 0.99865, which is close to the ideal value of 1, not only validates the high agreement and measurement accuracy between the phase demodulation results and the actual strain, but also confirms the feasibility of using the phase demodulation results as a reference for the cross-correlation algorithm, providing a reliable basis for high-precision optical fiber strain sensing in future applications.

**Figure 10:**
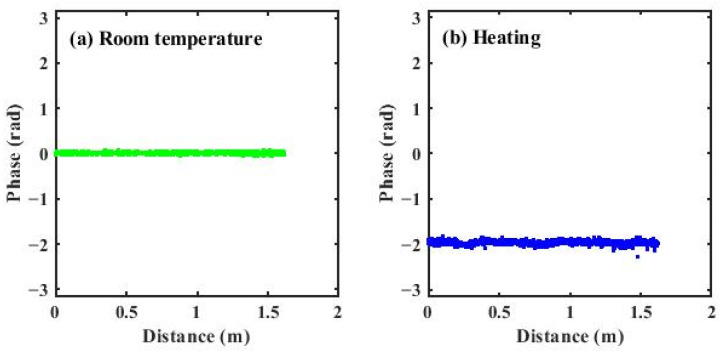
(**a**) phase demodulation under room-temperature conditions; (**b**) phase demodulation under heating conditions.

To investigate the influence of temperature drift on the phase demodulation results, a comparison was carried out between the phase distributions obtained at room temperature and those obtained under a uniform temperature increase of a few degrees. As shown in Figure 10, the phase distributions in the two cases exhibit nearly identical spatial profiles. The temperature variation only introduces a slowly varying and smooth background component. In both cases, the phase fluctuations are close to 0.15 rad. Therefore, the proposed method is robust against such moderate temperature drifts.

**Figure 11:**
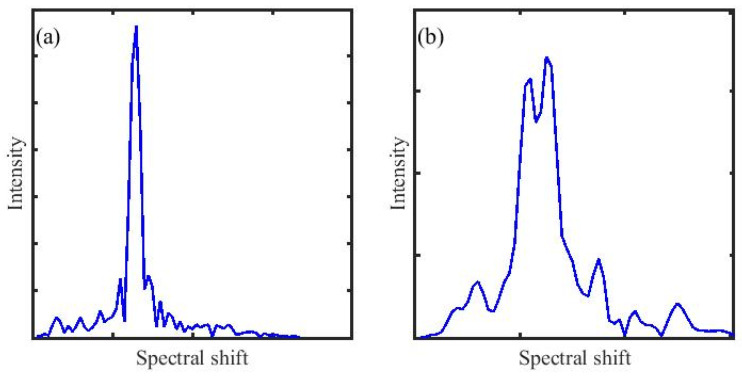
Cross-correlation results under different conditions: (**a**) in the absence of a strain burst, and (**b**) in the presence of a strain burst.

As shown in Figure 11a, when the strain within the correlation window is uniformly distributed, the reference signal and the measurement signal exhibit a high degree of similarity. The cross-correlation function between them shows a single, sharp main peak with a high amplitude. Only small sidelobes appear on both sides of the peak, indicating good signal matching within the analysis window and minimal local measurement error.

However, when the correlation window covers a strain discontinuity region, as illustrated in Figure 11b, the windowed signal segment simultaneously contains scattering components originating from different strain levels, which exhibit significantly different spectral characteristics. During the cross-correlation computation, these components with different spectral shifts produce different correlation maxima. Their superposition causes the originally single and concentrated main correlation peak to split into several closely spaced peaks. This results in peak distortion and a multi-peak structure, indicating a reduced degree of signal matching. As the main peak splits, the accuracy of the cross-correlation-based estimation deteriorates.

Guided by the strain boundary obtained from phase demodulation, the cross-correlation window is adaptively confined within signals from the same strain level. When the strain remains at a single level, the phase varies smoothly and a relatively large window is selected to ensure stable cross-correlation. When a strain discontinuity is present, the window width is automatically reduced near these locations. This avoids including scattering components from different strain levels and prevents the splitting of the correlation main peak. As a result, the cross-correlation window can be dynamically adjusted according to the phase features, enabling stable correlation results and reliable strain estimation near discontinuities.

**Figure 12:**
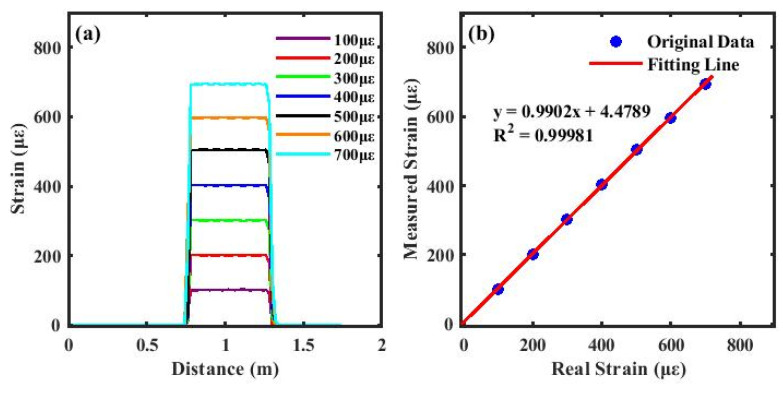
(**a**) Different strain measurements under cross-correlation; (**b**) linear fit between actual and measured strains.

The resulting strain distribution is presented in Figure 12a, where the spatial variation in strain along the fiber is clearly observable. In addition, the processing time of the proposed method is 0.035 s. According to the fitting result shown in Figure 12b, the intercept is 4.48 µε. This is mainly due to errors caused by noise. Such a fixed strain offset accounts for only a small fraction of the measured strain under large strain conditions. The figure also illustrates the linear relationship between the measured and actual strain values, exhibiting a high degree of linearity with a correlation coefficient R^2^ of 0.99981, which is very close to the ideal value of 1. This confirms the excellent performance of the adaptive-window-based cross-correlation method in both accurately locating strain regions and precisely mapping strain distributions. Moreover, the method effectively balances measurement resolution with computational efficiency, providing a robust technical foundation for high-resolution, high-precision optical fiber strain sensing applications. The demonstrated approach also offers a feasible and reliable reference for subsequent online monitoring in complex strain environments.

**Figure 13:**
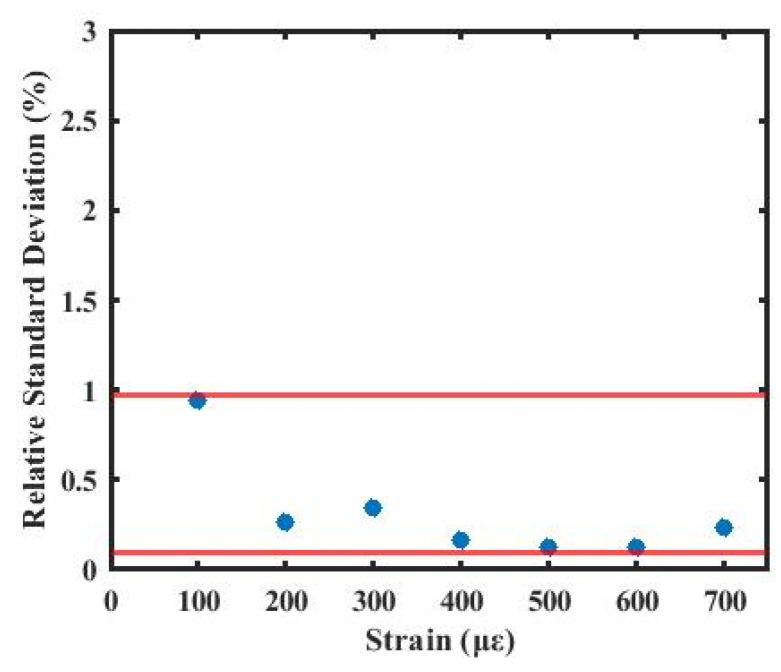
Measurement error at different spatial resolutions.

As shown in Figure 13, the relative standard deviation remains within the range of 0.13% to 0.94% over the applied strain range of 100–700 µε. The relative standard deviation at all these strain levels is below 1%, indicating good stability of the system under different strain conditions. These results further demonstrate that the proposed method enables precise and reliable distributed strain sensing.

## 4. Discussion

At very small strains, the strain-induced phase change approaches the noise floor, making it inaccurate when using the phase signal to locate the strained position. From Figure 9, we can estimate that the minimum detectable strain is approximate 30 µε. At very high strains, the phase difference between adjacent points becomes larger. When the phase difference between two adjacent points exceeds π, phase unwrapping failure occurs. In our experiment, the spatial distance between two sampling points is 0.27 mm. A strain of 1250 µε will induce a phase change of π, assuming the wavelength is 1550 nm and the refractive index is 1.467, according to Equation (7). In addition, noise can further increase the fluctuation of the phase demodulation result. So the maximum measurable strain is less than 1250 µε with the phase-OFDR method.

For smoothly varying strain distributions, the correlation window covers a specific signal length. And after normalization, the amplitude of the main correlation peak is reduced compared to that in the uniform-strain case. In this case, the measured result represents the average strain within the window.

For highly non-uniform strain distributions, phase demodulation provides high spatial resolution, enabling accurate localization of strain discontinuities. However, since the window cannot be made arbitrarily small, it may cross the boundary and lead to the multi-peak phenomenon shown in Figure 11. This issue will be further investigated in future work to optimize the window configuration and avoid the splitting of the main correlation peak.

## 5. Conclusions

In conclusion, this paper proposes a strain sensing method based on the fusion of phase demodulation and cross-correlation methods. The method enables accurate localization of strain occurrence with a spatial resolution of 0.27 mm. By combining the strain magnitude obtained through the cross-correlation algorithm, a complete strain distribution profile can be reconstructed. This approach preserves spatial resolution without requiring filtering operations. The experimental results demonstrate a strain linearity of 0.99981 within the 100–700 με strain range. Under the experimental setup with an Intel Core i7-14700KF and 32 GB RAM, the processing time of the proposed method is 0.035 s. These findings confirm that the proposed method provides an effective solution for distributed strain sensing, featuring high spatial resolution, fast processing speed, and low measurement error, while effectively addressing the issue of measurement inaccuracies caused by susceptibility to noise in conventional phase demodulation methods.

## Data Availability

Data can be provided by the corresponding author upon request.
